# Bioinformatics Approach to Identify the Influences of COVID-19 on Ischemic Stroke

**DOI:** 10.1007/s10528-023-10366-0

**Published:** 2023-05-15

**Authors:** Jiabao Zhu, Xiangui Li, Fanzhen Lv, Weimin Zhou

**Affiliations:** Department of Vascular Surgery, The Second Affliated Hospital of Nanchang University, Minde Road 1, Nanchang City, Jiangxi Province China

**Keywords:** COVID-19, Ischemic stroke, Thrombosis, Differentially expressed genes, Hub gene, Drug molecule

## Abstract

As severe acute respiratory syndrome coronavirus 2 (SARS-COV-2) is becoming more infectious and less virulent, symptoms beyond the lungs of the Coronavirus Disease 2019 (COVID-19) patients are a growing concern. Studies have found that the severity of COVID-19 patients is associated with an increased risk of ischemic stroke (IS); however, the underlying pathogenic mechanisms remain unknown. In this study, bioinformatics approaches were utilized to explore potential pathogenic mechanisms and predict potential drugs that may be useful in the treatment of COVID-19 and IS. The GSE152418 and GSE122709 datasets were downloaded from the GEO website to obtain the common differentially expressed genes (DEGs) of the two datasets for further functional enrichment, pathway analysis, and drug candidate prediction. A total of 80 common DEGs were identified in COVID-19 and IS datasets for GO and KEGG analysis. Next, the protein–protein interaction (PPI) network was constructed and hub genes were identified. Further, transcription factor–gene interactions and DEGs–miRNAs coregulatory network were investigated to explore their regulatory roles in disease. Finally, protein-drug interactions with common DEGs were analyzed to predict potential drugs. We successfully identified the top 10 hub genes that could serve as novel targeted therapies for COVID-19 and screened out some potential drugs for the treatment of COVID-19 and IS.

**Supplementary Information:**

The online version contains supplementary material available at 10.1007/s10528-023-10366-0.

## Introduction

The Coronavirus Disease 2019 (COVID-19) is an acute respiratory infection caused by SARS-COV-2 that has contributed to millions of deaths worldwide, with typical symptoms of fever, fatigue, dry cough, and also headache, hemoptysis, chest pain, and diarrhea (Hu et al. 2021). Previous study has shown that patients with SARS-COV-2 have increased levels of fibrinogen, D-dimer, and C-reactive protein, suggesting an increased risk of thrombosis, which is associated with poor prognosis (Han et al. 2020). The autopsy results in several COVID-19 patients indicated that not only macrovascular complications but also microvascular thrombosis were found in these patients (Carsana et al. 2020). The report implies a high association between COVID-19 and thrombosis. IS is the second leading cause of death worldwide, with an annual mortality rate of approximately 5.9 million, which is typically characterized by sudden onset of unilateral weakness, numbness, diplopia, slurred speech, ataxia, and nonorthogonal vertigo (Feigin et al. 2014).

After invading the human body, SARS-COV-2 is processed by transmembrane protease serine 2 (TMPRSS2), binds to angiotensin-converting enzyme 2 (ACE2), and enters host cells. ACE2, a part of the renin-angiotensin system (RAS), is expressed in airway epithelium, lung parenchyma, renal cells, heart, testis, vascular endothelial cells, intestinal epithelial cells and brain, and has significant antithrombotic effects by reducing platelet aggregation and nitric oxide release (Verdecchia et al. 2020). Angiotensin (1–7), the ACE2-mediated degradation product of angiotensin II, inhibits adhesion and migration of leukocytes in tissues and may act as an anti-inflammatory factor (Lelis et al. 2019). Dysregulation of ACE2 and Ang (1–7) levels may lead to thrombosis. During infection of vascular endothelial cells, SARS-COV-2 causes inflammatory cell infiltration, endothelial cell apoptosis, and elevation of pro-inflammatory cytokines (IL-1, IL-2, IL-6, IL-8, IL-10, IL-17, and TNF-α) through viral replication (Mehta et al. 2020). Some of these cytokines, such as IL-1, IL-6, and TNF-α, promote the release of tissue factor thereby activating exogenous clotting pathways. On the other hand, cytokines also promote NETs (neutrophil extracellular traps) formation, triggering extrinsic and intrinsic coagulation pathways that promote thrombin production. Correspondingly, NETs promote the release of inflammatory cytokines causing a cytokine storm (Hudock et al. 2020). In summary, such conditions may be extremely beneficial for blood hypercoagulability and thrombosis. One study has found that SARS-CoV-2 could infect cerebrovascular endothelial cells through the transcellular pathway to cross the blood–brain barrier, resulting in neuronal damage, which may increase the risk of in situ thrombosis. (Zhang et al. 2021a).

**Fig. 1:**
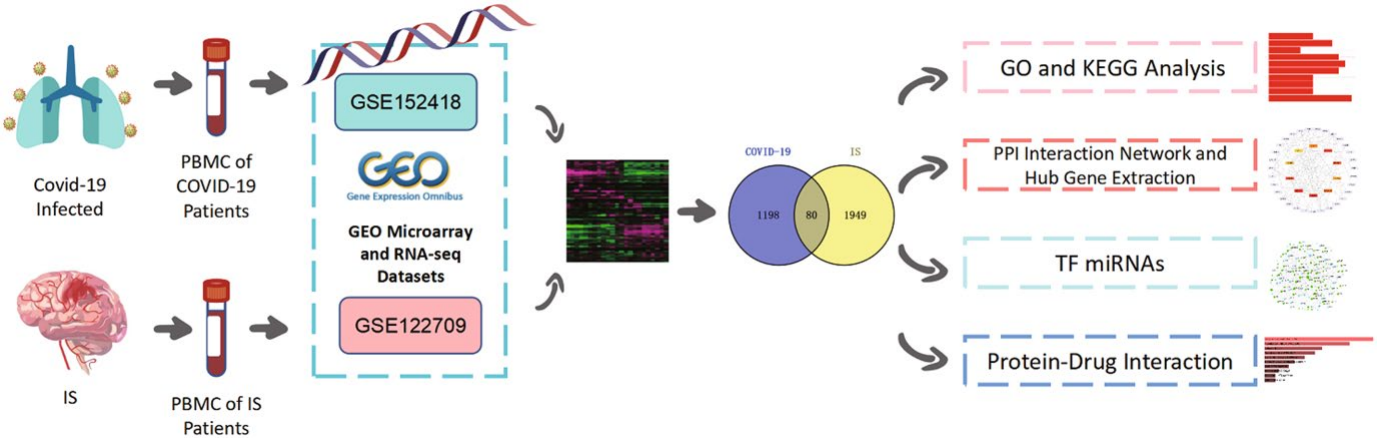
Schematic diagram of the total workflow in this study

In this study, two datasets, GSE152418 for COVID-19 and GSE122419 for IS, from Gene Expression Omnibus (GEO) database were analyzed to investigate the underlying molecular mechanisms of COVID-19 and IS through bioinformatics approaches. First, common differentially expressed genes (DEGs) were identified in these two datasets for GO enrichment analysis and KEGG pathway analysis. Next, a protein–protein interaction network (PPI) was constructed and hub genes were screened. Further, transcription factors (TFs) and miRNAs associated with DEGs were investigated. Finally, some potential drugs were predicted for the treatment of COVID-19 and IS that may help improve prognosis. The workflow of this study is shown in Fig. 1.

## Materials and Methods

### Data Collection

The gene expression datasets analyzed in this study were obtained from the GEO database of the National Center for Biotechnology Information (NCBI; https://www.ncbi.nlm.nih.gov/geo/). Microarray and RNA-seq datasets were downloaded to identify genes shared by COVID-19 and IS. The GEO accession ID for COVID-19 is GSE152418, including RNA-seq expression in peripheral blood mononuclear cells from 17 COVID-19 patients and 17 healthy controls sequenced on a high-throughput Illumina NovaSeq 6000 sequencer. Similarly, the GEO accession ID for IS is GSE122709, which contains RNA-seq expression profiling by HiSeq X Ten from peripheral blood mononuclear cells from 10 IS patients and 5 healthy control patients. The overall information of the database is shown in Table 1.

**Table 1:** Overview of GEO datasets characteristics and quantitative measures of COVID-19 and IS in this analysis

Disease name	GEO accession	GEO platform	Total DEGs count	Samples
COVID-19	GSE152418	GPL24676	1278	17 COVID-19:17 health
IS	GSE122709	GPL20795	2029	10 IS: 5 controls

### Identification of DEGs and Common DEGs Among COVID-19 and IS

Datasets were background corrected, normalized, and differentially analyzed using the Deseq2 package of R software (1.24.0). Differentially expressed genes were screened with *P* < 0.05 and ∣fold change (FC)∣ ≥ 2 as screening parameters. Then, the common DEGs for COVID-19 and IS were obtained using Jvenn, an online Venn analysis tool.

### Gene Ontology and Pathway Enrichment Analysis

Gene Ontology (GO) analysis and Kyoto Gene and Genome Database (KEGG) analysis were performed on the common DEGs using the R software package cluster Profiler, with *P* < 0.05 as the screening condition. GO analysis investigated the underlying biological processes, cellular components, and molecular functions of DEGs. KEGG is used to predict the role of protein interaction networks in various cellular activities.

### Protein–Protein Interaction Network Analysis

The list of common DEGs obtained above was submitted to the STRING online database (https://string-db.org/), and the minimum connection score was set to 0.4 to construct a protein interaction (PPI) network map. The MCC algorithm in the cytoHubba plugin of Cytoscape (v.3.9.1) was used to screen the top 10 genes in the PPI network as hub genes.

### Recognition of Transcription Factors and miRNAs Associated with Common DEGs

miRNAs–DEGs and transcription factor (TFs)–DEGs interaction network were identified to detect major changes at the transcriptional level. Using the NetworkAnalyst platform to find topology-reliable TFs that tend to bind to the common DEGs from the JASPAR database. miRNAs were extracted from the mirTarBase database to create miRNAs–DEGs network. Then, both miRNAs–DEGs and TFs–DEGs interaction networks were visualized in Cytoscape

### Evaluation of Applicant Drugs

Based on the common DEGs between COVID-19 and IS, we used the Drug Signatures database (DSigDB) via the Enrichr web platform to identify drug molecules. DSigDB is a novel gene library based on data on quantitative inhibition and drug-induced changes in gene expression by drugs and compounds.

### Gene–Disease Association Analysis

DisGeNET is a library of disease-related genes that can be used for a variety of research purposes, including molecular studies of specific human diseases and their complications, characterization of causative genes, hypotheses about the efficacy and side effects of drug treatments, disease candidate gene identification and validation, and performance evaluation of text mining techniques. We further explored the relationship between genes and diseases through NetworkAnalyst platform.

## Results

### Identification of DEGs and Common DEGs Between IS and COVID-19

**Fig. 2:**
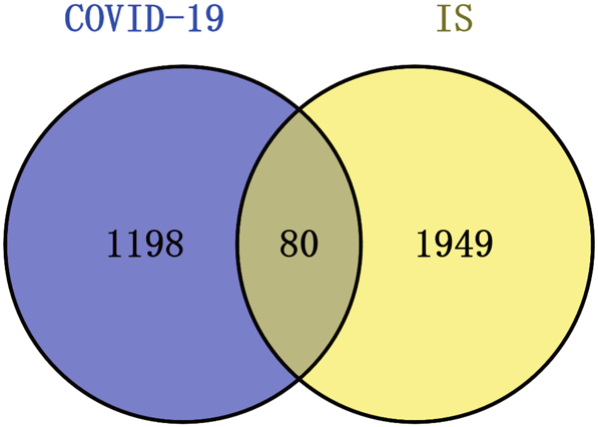
This study included a microarray and an RNA-seq dataset for IS (GSE122709) and COVID-19 (GSE152418). The analysis showed that IS and COVID-19 shared 80 common DEGs

To investigate the relationship between IS and COVID-19, we obtained the corresponding human RNA-seq dataset and microarray dataset from the GEO database and then performed data analysis using the DESeq2 and limma packages of R software. *P* < 0.05 and ∣fold change (FC)∣ ≥ 2 as screening parameters. In this study, 1278 differential expression genes were obtained in the COVID-19 dataset, including 19 up-regulated genes and 1259 down-regulated genes, while 2229 differential expression genes were obtained in the IS dataset, including 740 up-regulated genes and 1289 down-regulated genes. 80 common DEGs was identified via the Jvenn website in the two datasets for further analysis. Figure 2 shows the common DEGs of the two datasets.

### Gene Ontology and Pathway Enrichment Analyses

**Fig. 3:**
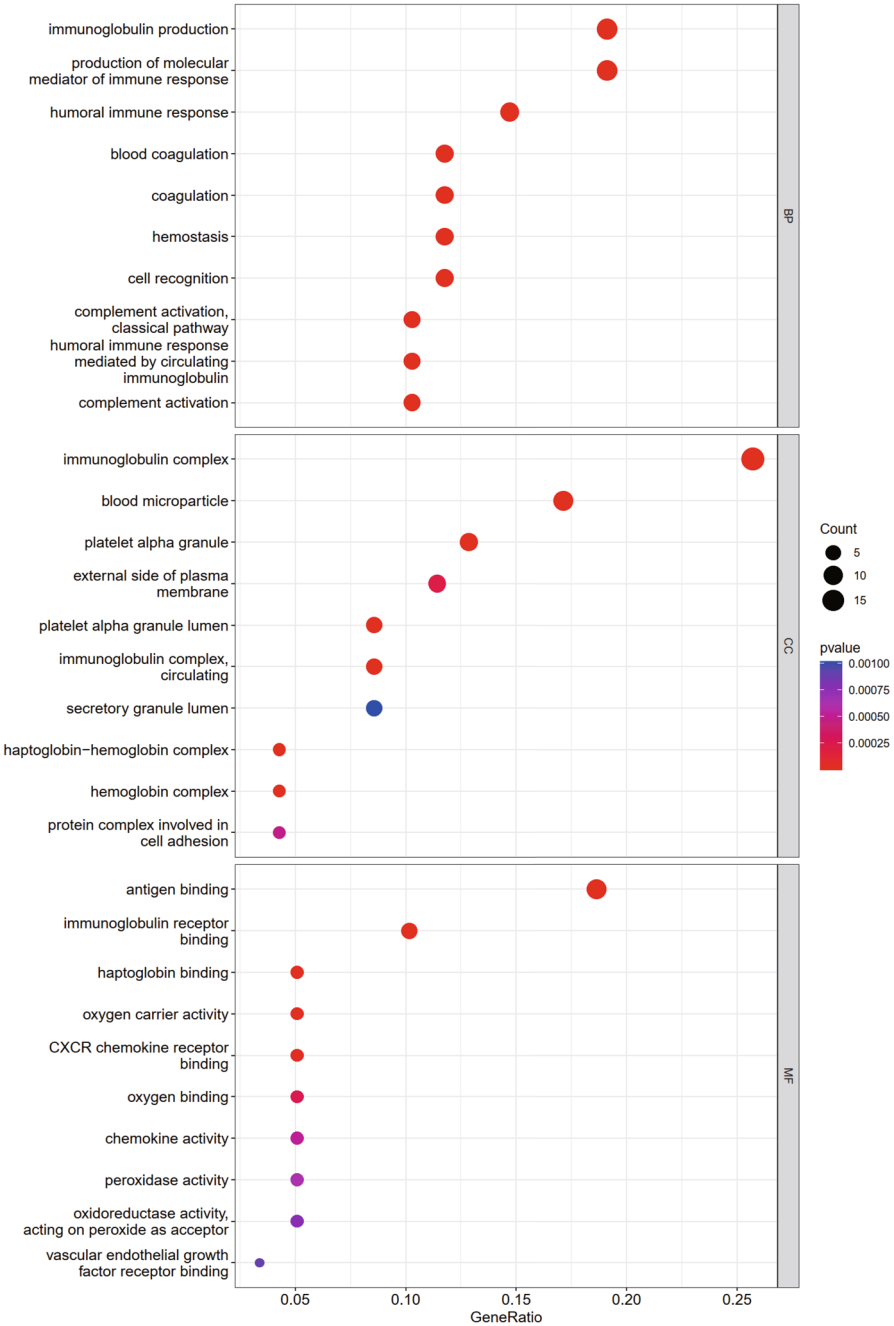
Bubble graphs of ontological analysis of the DEGs between COVID-19 and IS, including BP: biological processes, CC: molecular function, and MF: cellular component

**Fig. 4:**
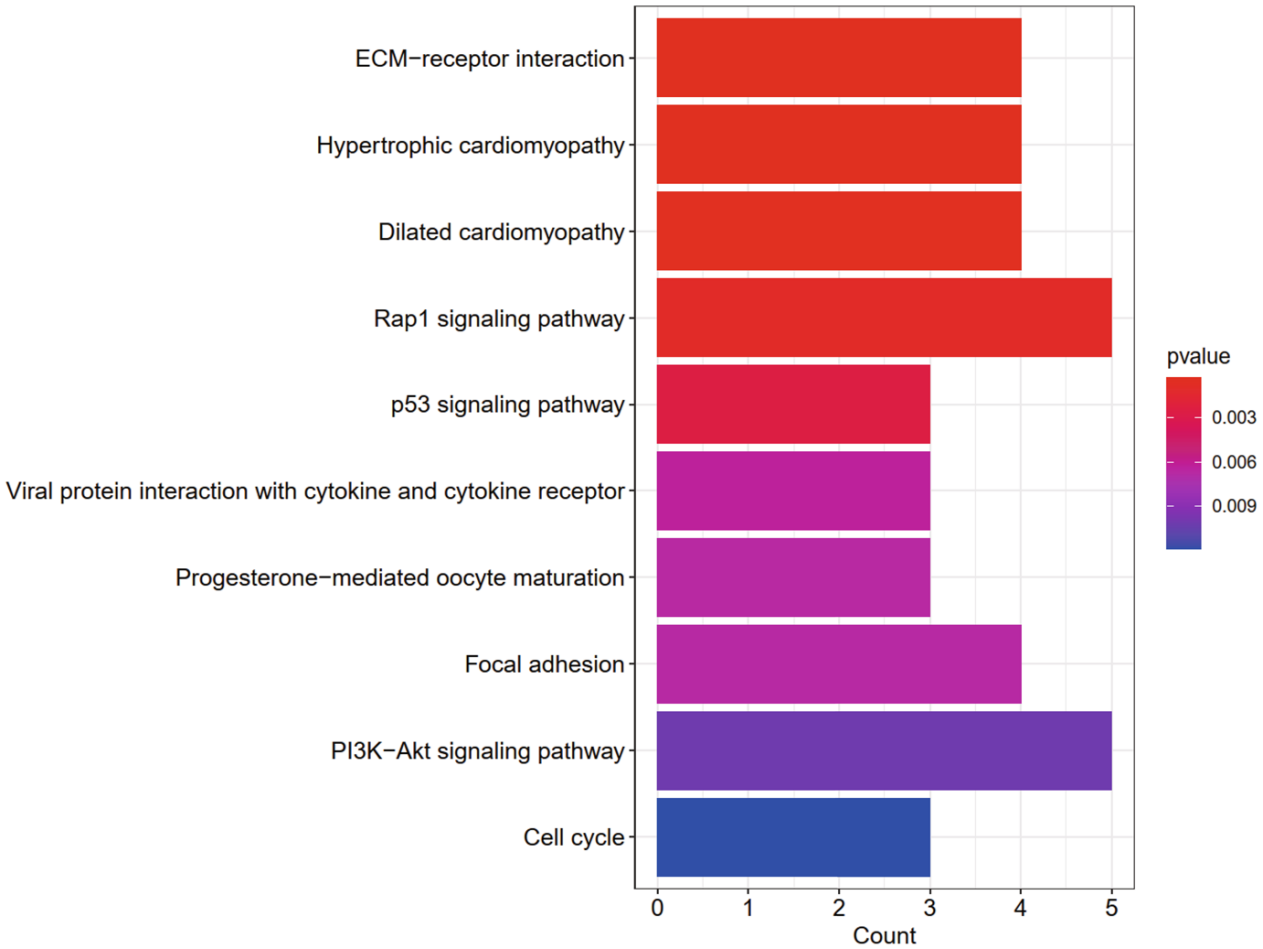
The bar graphs of KEGG pathway enrichment analysis of the DEGs between COVID-19 and IS

Gene function and pathway enrichment analyses were performed using R to determine the biological significance and enrichment pathways of the common DEGs in this study. Gene ontology was obtained in three categories (biological processes, cellular components, and molecular functions), and the GO database was selected as the annotation source. The top 10 terms in categories of biological processes, molecular functions, and cellular components are summarized in Table S1. Figure 3 also characterizes the overall ontological analysis linearly for each category in the bubble graph. GO ontology of common DEGs showed that the common DEGs were mainly concentrated in immunoglobulin production, production of immune response molecular mediators, complement activation, classical pathway, and humoral immune response. KEGG analysis of common DEGs are mainly concentrated in the following signaling pathways: ECM receptor interaction, Rap1 signaling pathway, p53 signaling pathway, and the interaction of viral proteins with cytokines and cytokine receptors. Table S2 shows the KEGG enrichment paths derived from the selected dataset. For a more precise description, Fig. 4 also presents the pathway enrichment analysis by a bar graphs.

### Classification of Hub Proteins and Submodule

**Fig. 5:**
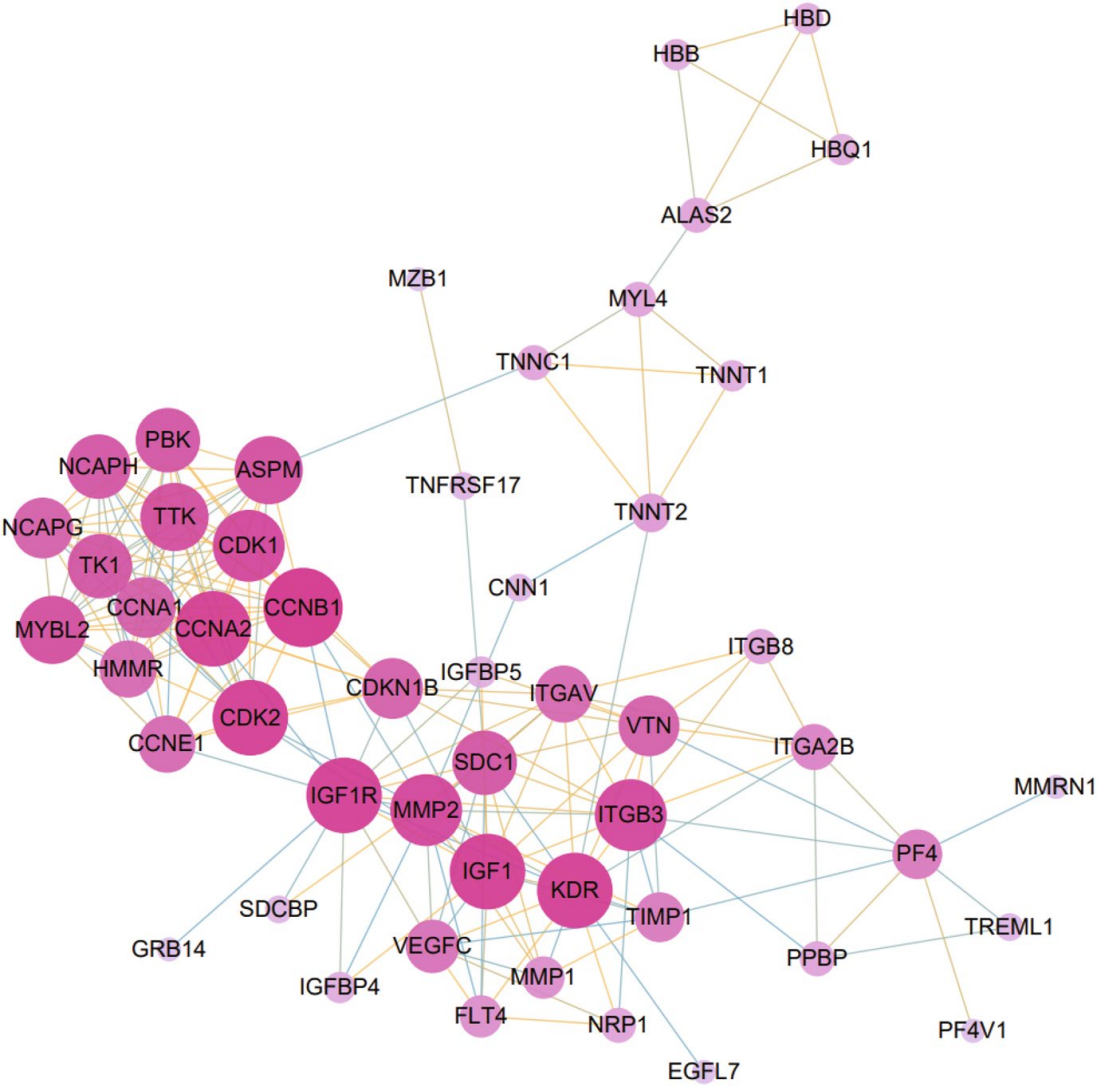
The PPI network of the common DEG of COVID-19 and IS, containing 51 nodes and 199 edges, was generated using String and visualized in Cytoscape

**Fig. 6:**
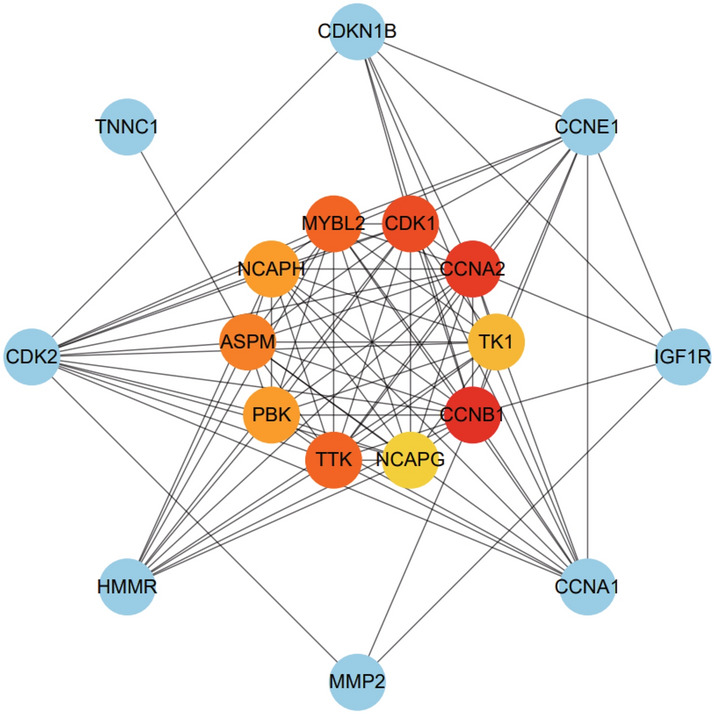
Identification of hub genes from PPI networks using Cytohubba plugin in Cytoscape. Here, the colored nodes in the inner circle represent the hub genes (Color figure online)

We scrutinized protein–protein interaction (PPI) network from STRING and visualized in Cytoscape to anticipate common DEGs’ interactions and adhesion pathways. Taking the combined score > 0.4 as the criterion to remove isolated nodes, the results show that the PPI network consists of 51 nodes and 199 edges (Fig. 5). The top 10 hub genes were calculated by the Cytohubba plugin in Cytoscape. The hub genes were ranked according to the MCC algorithm: CCNB1, CCNA2, CDK1, TTK, MYBL2, ASPM, NCAPH, PBK, TK1, and NCAPG. These hub genes may be potential biomarkers and may also lead to new treatment strategies for investigated diseases. A submodule network was created using the Cytohubba plugin to better understand their close association and proximity. The extended network of hub gene interactions obtained from the PPI network is presented in Fig. 6.

### Construction of Regulatory Signatures

**Fig. 7:**
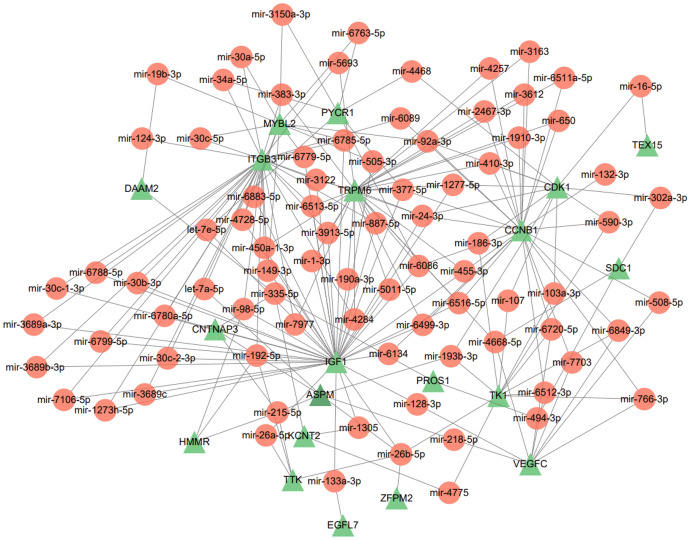
Interaction network of DEG–miRNAs obtained from the NetworkAnalyst. Here, the orange circular nodes represent miRNAs and the green triangular nodes as gene symbols interact with miRNAs (Color figure online)

**Fig. 8:**
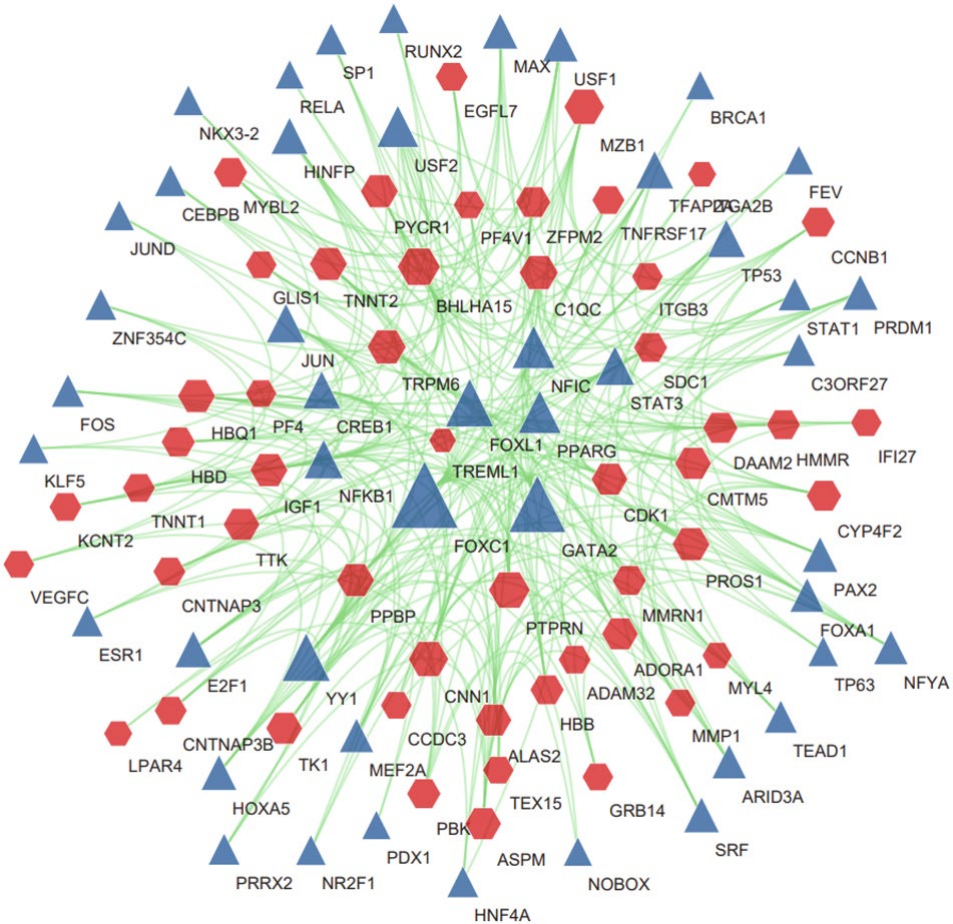
Interaction network of DEG–TFs obtained from NetworkAnalyst. Here, the blue triangle nodes represent TFs and the red hexagonal nodes represent genes interacting with TFs (Color figure online)

To identify the occurring changes at the transcriptional level, we used a network-based approach to decode regulatory TFs and miRNAs to understand the key molecules regulating DEGs. The interaction of miRNAs regulators with common DEGs is shown in Fig. 7. Likewise, Fig. 8 shows the interaction of TFs regulators with common DEGs.

### Identification of Candidate Drugs

Identification of protein–drug interactions is important for understanding the proposed structural features of receptor sensitivity. Based on the common DEGs of IS and COVID-19, we identified 9 possible compounds from the DSigDB database. The top 9 compounds were Phytoestrogens, Lucanthone, 58-64-0, genistein, N-Methyl-N-nitrosourea, deferoxamine, monobenzone, ethylene dimethacrylate, and ciclopirox. Table 2 shows more details of the predicted compounds.

**Table 2:** List of potential therapeutic drugs for COVID-19

Term	*P*-value	Genes	Structure
Phytoestrogens CTD 00007437	4.13E−08	ASPM; PBK; CDK1; MYBL2; TTK; TK1	
LUCANTHONE CTD 00006227	2.26E−06	ASPM; CCNB1; MMP1; PBK; CDK1; MYBL2; TTK; HMMR	
58-64-0 CTD 00005321	5.22E−06	ITGB3; ITGA2B; PPBP; PF4	
Genistein CTD 00007324	5.95E−06	MMP1; PROS1; ITGB3; CYP4F2; VEGFC; TTK; BHLHA15; IGF1; ASPM; CNN1; CCNB1; TNNT1; PBK; CDK1; MYBL2; TK1; ZFPM2	
*N*-Methyl-*N*-nitrosourea CTD 00006319	3.32E−05	CDK1; HBB; IGF1	
Deferoxamine MCF7 DOWN	6.69E−05	ASPM; CCNB1; HMMR	
Monobenzone MCF7 DOWN	7.78E−05	ASPM; CCNB1; HMMR	
Ethylene dimethacrylate BOSS	1.11E−04	MMRN1; MMP1; HBB; HMMR; IGF1	
Ciclopirox MCF7 DOWN	1.50E−04	ASPM; CCNB1; TTK; HMMR	

### Identification of Disease Association

**Fig. 9:**
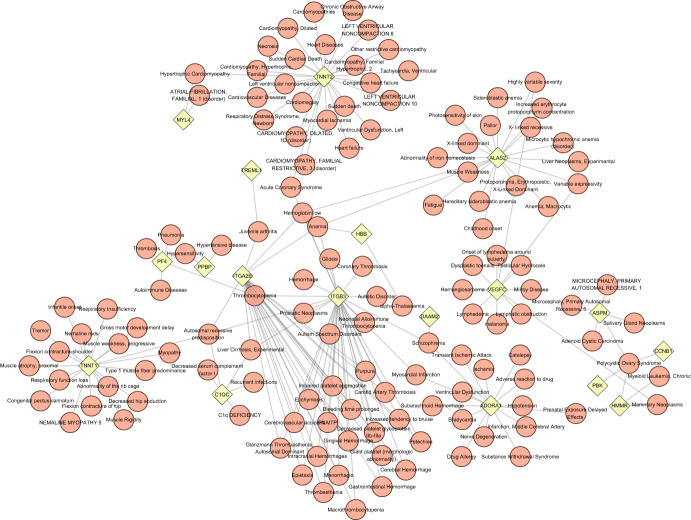
The Gene–Disease association network indicates diseases connected with common DEGs. Diamond-shaped points indicate genes and the circular nodes refer to diseases associated with genes

The association between different diseases is usually by having one or more similar genes. Treatment design strategies for disease often begin by uncovering the relationship between genes and disease. Through NetworkAnalyst’s analysis of gene–disease associations, we noted that Anemia, Polycystic Ovary Syndrome, Hemoglobin low, Schizophrenia, and Thrombocytopenia was associated with our reported genes are the most coordinated. The association between genes and diseases is shown in Fig. 9.

## Discussion

Ischemic stroke refers to the occlusion or severe stenosis of cerebral blood vessels in the brain caused by damage to cerebral blood flow, resulting in a decrease in cerebral blood flow and brain tissue necrosis in the cerebral blood supply area. COVID-19 is a global public health emergency, and the pathogen SARS-CoV-2 can trigger an inflammatory storm that causes damage to the body. In this article, we examined the DEGs in COVID-19 and IS to investigate possible links between them.

In this study, we analyzed the gene expression profiles of COVID-19 and IS separately to obtained common DEGs and then characterized the functions of DEGs using GO and KEGG enrichment analyses. As for GO analysis, the GO terms were mainly associated with immune- and coagulation-related processes. It was shown (Han et al. 2020) that SARS-CoV-2 induces a systemic hypercoagulable state with elevated levels of D-dimer and fibrinogen, which are susceptibility factors for IS. In addition, elevated cytokine levels, endothelial cell damage, platelet activation, and complement activation are potential mechanisms of hypercoagulability in COVID-19 patients. The cytokine storm caused by COVID-19 can damage endothelial cells, upregulate tissue factor expression, activate extrinsic coagulation pathways, and contribute to thrombosis. Moreover, damaged endothelial cells may inhibit the fibrinolytic system and enhance the expression of PAI-1 (Zhang et al. 2021b). In the KEGG analysis, the 10 major KEGG signaling pathways are ECM–receptor interaction, Hypertrophic cardiomyopathy, Dilated cardiomyopathy, Rap1 signaling pathway, p53 signaling pathway, Viral protein interaction with cytokine and cytokine receptor, Progesterone-mediated oocyte maturation, Focal adhesion, PI3K-Akt signaling pathway, and Cell cycle. One study (Kumar et al. 2022) has found that overexpression of p53 significantly reduced COVID-19 virus production, suggesting that p53 is a host innate antiviral immunity that limits SARS-CoV-2 replication. The PI3K/AKT signaling pathway plays a key role in cell proliferation, survival, growth, migration, invasion, and other processes, which also inhibits apoptosis and promotes angiogenesis. Furthermore, PI3K/Akt/mTOR inhibitors control cellular activation in SARS-CoV-2 infection and may have antiviral effects (Basile et al. 2022).

PPI network was created based on DEGs genes to understand the mutual biological characteristics of proteins and further predict therapeutic targets. According to the MCC method, the top 10 hub genes are CCNB1, CCNA2, CDK1, TTK, MYBL2, ASPM, NCAPH, PBK, TK1, and NCAPG. Cell cycle progression depends on cyclins and cell cycle-dependent kinases. Previous studies have shown that CCNB1 binds to another hub gene, CDK1, to form a cyclinB1/CDK1 complex, which coordinates cell cycle G2/M progression, increases mitochondrial respiration, and promotes mitosis (Hochegger et al. 2008). In IS, accumulation of the cyclin B1/CDK1 complex in mitochondria leads to oxidative stress and energy deficit and neuronal apoptosis. Meanwhile, it has been found that the high inflammatory state and oxidative stress in COVID-19 patients lead to mitochondrial dysfunction and cellular DNA damage. Again, as mitochondrial dysfunction and cellular DNA damage are associated with cell cycle protein B1 activity (Saleh et al. 2020; Wang et al. 2014). Therefore, in this context, dysregulation of cyclin B1/CDK1 complex activity may be one of the mechanisms by which patients with COVID-19 are complicated with IS and affect the prognosis. Cyclin A2 (CCNA2) is also an essential protein for cell cycle progression and is responsible for the continuation of the S phase and entry into mitosis together with CDK1 or CDK2. Cyclin A2 is required for the activation and nuclear accumulation of cyclin B1/CDK1. Therefore, Cyclin A2 may be another potential target for the treatment of COVID-19 and IS through the mechanism of affecting the level of cyclin B1/CDK1. TTK, a human homologous kinase of yeast MPS1, is activated by Aurora B and cyclin B1/CDK1 during early mitosis, it has been shown that cyclin B1/CDK1 required to ensure mitotic fidelity by enabling MPS1 kinetochore localization (Hayward et al. 2019). MYBL2 (B-MYB), one of the MYB family of transcription factors involved in cell cycle progression, has been proved to participates in cell senescence and apoptosis through multiple mechanisms (Mowla et al. 2014), such as in vascular endothelial cells, down-regulation of B-MYB promotes senescence through the ROS-mediated p53/p21 pathway (Zhou et al. 2017). ASPM (abnormal spindle-like microcephaly), also known as MCPH5, is mainly distributed between centrosomes and spindle microtubules, and the absence of ASPM results in the disruption of spindle assembly and mitotic processes. ASPM mutations are the most common cause of primary microcephaly in humans. In additional, ASPM may promote the progression of prostate cancer and hepatocellular carcinoma by enhancing the Wnt/β-catenin signaling pathway (Pai et al. 2019). NCAPH (non-SMC condensing I complex subunit H) is one of the constituent structures of condensing I complex, which also includes NCAPG (non-SMC condensin I complex subunit I), NCAPD2 (non-SMC condensing I complex subunit D2), and SMC (structural maintenance of chromosomes). NCAPH and NCAPG are closely related to cancer. NCAPH inhibits phosphorylation of β-catenin by forming a complex with β-catenin and activating Wnt signaling to promote the formation of non-small cell lung cancer (Xiong et al. 2021). PBK (PDZ-binding kinase) is a mitotically active Serine/Threonine Kinase, also referred to as T-lymphokine-activated killer-cell-originated protein kinase (TOPK). There is research showing that PBK inhibition aggravates H_2_O_2_-induced cardiomyocyte oxidative stress, while overexpression of PBK has the opposite effect through positive regulation of the ERK pathway (Sun et al. 2016).

Next, we analyzed TFs–DEGs and miRNAs–DEGs interaction networks. TFs can coordinate gene transcription, while miRNAs mainly regulate the expression of target mRNAs through different pathways. In our analysis, FOXC1, GATA2, YY1, and PPARG may play a role in the development of COVID-19 and IS. Upregulation of FOXC1 resists apoptosis and inflammation in eye disease and chronic obstructive pulmonary disease (COPD) (Berry et al. 2008). FOXC1 also improve neurological dysfunction in IS and affect the self-renewal and proliferation of arachnoid-pia stem cells (APSCs) by regulating STI-1/PrP^C^ signaling (Lee et al. 2019). Enhanced activity of GATA2 under the influence of IL1β induces TMPRSS2 expression and increases susceptibility to SARS-COV-2 infection (Cioccarelli et al. 2021). In the cerebral ischemia/reperfusion (CI/R) model, YY1 binds to lncRNA GAS5 to form a complex that fosters PFKFB3 transcription, glycolysis, and neuronal apoptosis (Zhang et al. 2019). Activation of PPARG can effectively reduce neuronal oxidative stress and inflammatory processes and protect brain tissue from hypoxia and ischemia (Zhao et al. 2009), and the down-regulation of PPARG expression is highly correlated with the over-activation of β-catenin signaling. On the other hand, ACE2 expression level has been inversely correlated with the Wnt/β-catenin signaling pathway. Through these links, it is suggested that PPARG agonists may be potential agents for the treatment of SARS-COV-2 and IS by influencing the Wnt/β-catenin signaling pathway and ACE2 expression levels. In miRNAs–DEGs, overexpression of miR-26b-5p, miR-24-3p, and miR-98-5p has been confirmed to improve CI/ R model ischemic injury caused by ischemic injury, cell inflammation, and apoptosis (Shangguan et al. 2020; Zhou et al. 2022; Di et al. 2021). MiR-98-5p affects SARS-COV-2 entry into host cells by inhibiting mRNA expression of TMPRSS2 (Wang et al. 2021).

Several candidate small-molecule compounds and drugs have been used as therapeutic agents for COVID-19, most of which exert antiviral effects by inhibiting specific steps in the viral life cycle. For example, Umifenovir and Camostat (Breining et al. 2021) mesylate both exert antiviral effects by preventing the virus from entering cells. Chloroquine and hydroxychloroquine (Meo et al. 2020), commonly used anti-malarial drugs in clinical practice, can exert antiviral effects through mechanisms of action, such as inhibition of membrane fusion, glycosylation, host receptors, endocytosis, and reduction of cytokine production, which are potential drugs for the treatment of SARS-CoV-2. In addition, lopinavir (Cao et al. 2020), a protease inhibitor of HIV, has been found to exert antiviral effects by inhibiting SARS-CoV-2-associated 3-chymotrypsin-like protease. Similarly, favipiravir, ribavirin, and raltegravir prevent the formation of viral structural proteins by inhibiting RNA-dependent RNA polymerase (Rehman et al. 2021). In this study, we predicted a variety of drugs that may be effective for the treatment of SARS-CoV-2. Phytoestrogens, similar to estrogens in animals, can bind to estrogen receptors and enter the cell nucleus, affecting many physiological and pathological processes, such as reproduction, skin aging, bone, cardiovascular, nervous system, immune system, metabolism, and cancer (Rietjens et al. 2017). Desferrioxamine (van der Loo et al. 2020), one of the most widely used iron chelators in clinical practice, can binds free iron or aluminum in the blood and increases their excretion in the urine, reducing damage to various organs and tissues from excess iron and aluminum ions in the body. Lucanthone, a novel inhibitor of autophagy, induces histone D-mediated apoptosis and improves the prognosis of breast cancer (Carew et al. 2011). The results of our analysis suggested that the above drugs may be potential agents for the treatment of COVID-19.

We also performed gene–disease (GD) analysis to predict the association of DEGs and different diseases with degree more than 1. We found that the diseases associated with COVID-19 include stroke, cardiovascular diseases, and blood system diseases. In the literature (Shi et al. 2020), some patients with COVID-19 show signs of myocardial injury, such as elevated serum cardiac biomarker levels or abnormal electrocardiograms and echocardiograms, and the occurrence of myocardial infarction is associated with higher in-hospital mortality. In addition, arrhythmias (Wang et al. 2020) are not uncommon in patients with COVID-19, especially in the intensive care unit. Heart failure (Mehra and Ruschitzka 2020) is also a common complication in patients with COVID-19 and may be associated with cardiac diastolic dysfunction, chronic vascular disease, and SARS-CoV-2-induced sepsis.

## Conclusion

We successfully identified the top 10 hub genes that could serve as novel targeted therapies for COVID-19 and screened out some potential drugs for the treatment of COVID-19 and IS.

### Supplementary Information

Below is the link to the electronic supplementary material.

Supplementary file 1 (PDF 237 KB)

## Data Availability

The datasets GSE152418 and GSE122709 for this study can be found in the Gene Expression Omnibus (https://www.ncbi.nlm.nih.gov/geo/).
