# Supplementary material for: Bioinformatics Approach to Identify the Influences of COVID-19 on Ischemic Stroke
Source: Biochem Genet. 2023 May 15;61(6):2222–41. doi: 10.1007/s10528-023-10366-0 (PMC10184096; doi:10.1007/s10528-023-10366-0)
Supplement: Supplementary file 1 — (PDF 237 KB) [file 10528_2023_10366_MOESM1_ESM.pdf]

# Bioinformatics Approach to Identify the Influences of COVID-19 on Ischemic Stroke

Jiabao Zhu[1†], Xiangui Li[1†], Fanzhen Lv[1], Weimin Zhou[1*]

[1]Department of Vascular Surgery, The Second Affiliated Hospital of Nanchang University, Nanchang, Jiangxi, China

[†] These authors contributed equally to this work.

***Correspondence:**
Weimin Zhou, Department of Vascular Surgery, The Second Affliated Hospital of Nanchang University, Minde Road 1, Nanchang City, Jiangxi Province, China. drzwm@sina.com

## Supplementary Information (SI)

| ONTOLOGY | ID | Description | pvalue | geneID |
|---|---|---|---|---|
| BP | GO:0002377 | immunoglobulin production | 8.56E-13 | IGKV2-29/IGKV2D-30/IGKV3D-11/IGKV1D-8/MZB1/IGLV4-69/IGLV9-49/IGLV1-47/IGLV7-46/IGLV7-43/IGLV1-36/IGLV3-27/IGLV3-25 |
| | GO:0002440 | production of molecular mediator of immune response | 7.81E-11 | IGKV2-29/IGKV2D-30/IGKV3D-11/IGKV1D-8/MZB1/IGLV4-69/IGLV9-49/IGLV1-47/IGLV7-46/IGLV7-43/IGLV1-36/IGLV3-27/IGLV3-25 |
| | GO:0006958 | complement activation, classical pathway | 1.23E-07 | C1QC/IGHA2/IGHG4/IGHV3-20/IGLC2/IGLC3/IGLC7 |
| | GO:0006959 | humoral immune response | 1.97E-07 | C1QC/PF4V1/PF4/PPBP/IGHA2/IGHG4/IGHV3-20/IGLC2/IGLC3/IGLC7 |
| | GO:0002455 | humoral immune response mediated by circulating immunoglobulin | 2.69E-07 | C1QC/IGHA2/IGHG4/IGHV3-20/IGLC2/IGLC3/IGLC7 |
| | GO:0006956 | complement activation | 4.62E-07 | C1QC/IGHA2/IGHG4/IGHV3-20/IGLC2/IGLC3/IGLC7 |
| | GO:0007596 | blood coagulation | 1.30E-06 | PROS1/PF4V1/PF4/MMRN1/TREML1/HBB/ITGB3/CYP4F2 |
| | GO:0050817 | coagulation | 1.53E-06 | PROS1/PF4V1/PF4/MMRN1/TREML1/HBB/ITGB3/CYP4F2 |
| | GO:0007599 | hemostasis | 1.58E-06 | PROS1/PF4V1/PF4/MMRN1/TREML1/HBB/ITGB3/CYP4F2 |
| | GO:0008037 | cell recognition | 1.64E-06 | ADAM32/CNTNAP3/IGHA2/IGHG4/IGHV3-20/IGLC2/IGLC3/IGLC7 |
| CC | GO:0019814 | immunoglobulin complex | 3.49E-22 | IGKV2-29/IGKV2D-30/IGKV3D-11/IGKV1D-8/IGHA2/IGHG4/IGHV3-20/IGLV4-69/IGLV9-49/IGLV1-47/IGLV7-46/IGLV7-43/IGLV1-36/IGLV3-27/IGLV3-25/IGLC2/IGLC3/IGLC7 |
| | GO:0072562 | blood microparticle | 1.48E-13 | C1QC/IGKV3D-11/PROS1/HBB/HBD/IGHA2/IGHG4/ITGA2B/IGLV1-47/IGLV3-25/IGLC2/IGLC3 |
| | GO:0031091 | platelet alpha granule | 3.46E-11 | PROS1/PF4/PPBP/MMRN1/VEGFC/TREML1/IGF1/ITGA2B/ITGB3 |
| | GO:0031093 | platelet alpha granule lumen | 1.40E-07 | PROS1/PF4/PPBP/MMRN1/VEGFC/IGF1 |
| | GO:0042571 | immunoglobulin complex, circulating | 3.24E-07 | IGHA2/IGHG4/IGHV3-20/IGLC2/IGLC3/IGLC7 |

| | | | | |
|---|---|---|---|---|
| | GO:0031838 | haptoglobin-hemoglobin complex | 7.06E-06 | HBB/HBD/HBQ1 |
| | GO:0005833 | hemoglobin complex | 9.39E-06 | HBB/HBD/HBQ1 |
| | GO:0009897 | external side of plasma membrane | 0.000213 | SDC1/IGHA2/IGHG4/IGHV3-20/ITGA2B/IGLC2/IGLC3/IGLC7 |
| | GO:0098636 | protein complex involved in cell adhesion | 0.000486 | MMRN1/ITGA2B/ITGB3 |
| | GO:0034774 | secretory granule lumen | 0.001019 | PROS1/PF4/PPBP/MMRN1/VEGFC/IGF1 |
| MF | GO:0003823 | antigen binding | 7.39E-12 | IGKV3D-11/IGHA2/IGHG4/IGHV3-20/IGLV1-47/IGLV7-43/IGLV3-27/IGLV3-25/IGLC2/IGLC3/IGLC7 |
| | GO:0034987 | immunoglobulin receptor binding | 2.09E-07 | IGHA2/IGHG4/IGHV3-20/IGLC2/IGLC3/IGLC7 |
| | GO:0031720 | haptoglobin binding | 3.69E-06 | HBB/HBD/HBQ1 |
| | GO:0005344 | oxygen carrier activity | 1.11E-05 | HBB/HBD/HBQ1 |
| | GO:0045236 | CXCR chemokine receptor binding | 2.47E-05 | PF4V1/PF4/PPBP |
| | GO:0019825 | oxygen binding | 0.000263 | HBB/HBD/HBQ1 |
| | GO:0008009 | chemokine activity | 0.000519 | PF4V1/PF4/PPBP |
| | GO:0004601 | peroxidase activity | 0.000618 | HBB/HBD/HBQ1 |
| | GO:0016684 | oxidoreductase activity, acting on peroxide as acceptor | 0.000768 | HBB/HBD/HBQ1 |
| | GO:0005172 | vascular endothelial growth factor receptor binding | 0.000896 | VEGFC/ITGB3 |

**Table S1** Ontological analysis of common DEGs among SARS-CoV-2 and IS

Note: Top 10 terms of each category are listed

**Table S2** Pathway enrichment analysis of common DEGs among SARS-CoV-2 and IS

| ID | Description | p-value | geneID |
|---|---|---|---|
| hsa04512 | ECM-receptor interaction | 0.000321 | SDC1/HMMR/ITGA2B/ITGB3 |
| hsa05410 | Hypertrophic cardiomyopathy | 0.000349 | TNNT2/IGF1/ITGA2B/ITGB3 |
| hsa05414 | Dilated cardiomyopathy | 0.000447 | TNNT2/IGF1/ITGA2B/ITGB3 |
| hsa04015 | Rap1 signaling pathway | 0.001069 | VEGFC/LPAR4/IGF1/ITGA2B/ITGB3 |
| hsa04115 | p53 signaling pathway | 0.002594 | CCNB1/CDK1/IGF1 |
| hsa04061 | Viral protein interaction with cytokine and cytokine receptor | 0.006293 | PF4V1/PF4/PPBP |
| hsa04914 | Progesterone-mediated oocyte maturation | 0.006648 | CCNB1/CDK1/IGF1 |
| hsa04510 | Focal adhesion | 0.006723 | VEGFC/IGF1/ITGA2B/ITGB3 |
| hsa04151 | PI3K-Akt signaling pathway | 0.010086 | VEGFC/LPAR4/IGF1/ITGA2B/ITGB3 |
| hsa04110 | Cell cycle | 0.011852 | CCNB1/TTK/CDK1 |

**Table S3** the fold change of common DEGs in COVID-19 and IS, respectively

| Gene symbol | COVID-19 | IS |
|---|---|---|
| ADAM32 | 2.25519 | 2.644808 |
| ADORA1 | -2.48698 | 2.981777 |
| ALAS2 | 6.285087 | 2.198292 |
| ASPM | 2.89359 | 2.478286 |

| | | |
|---|---|---|
| BHLHA15 | 3.508463 | 3.059393 |
| C1QC | 3.480919 | 2.411934 |
| CCDC3 | 2.003099 | 3.915235 |
| CCNB1 | 2.212427 | 2.06743 |
| CDK1 | 2.652926 | 2.470697 |
| CMTM5 | 2.751215 | 2.797343 |
| CNN1 | 2.833126 | 2.719503 |
| CNTNAP3 | 2.163199 | -3.35391 |
| CNTNAP3B | 2.732845 | -3.06962 |
| CYP4F2 | 3.042319 | -4.27293 |
| DAAM2 | 2.498734 | -4.98126 |
| EGFL7 | 2.413626 | 2.408942 |
| FLG-AS1 | 2.068455 | 3.549604 |
| GLIS1 | 2.16379 | -2.37103 |
| GRB14 | 2.774111 | 2.121552 |
| HBB | 4.838255 | 2.022102 |
| HBD | 6.3858 | 3.125742 |
| HBQ1 | 2.811 | 2.352905 |
| HMMR | 2.853816 | 2.557774 |
| IFI27 | 8.638309 | 3.227019 |
| IGF1 | 4.068936 | 2.293879 |

| | | |
|---|---|---|
| IGHA2 | 3.335309 | 2.18739 |
| IGHG4 | 2.570169 | 2.978381 |
| IGHV3-20 | 4.761924 | 2.39249 |
| IGHV3-52 | 3.659518 | 2.784599 |
| IGKV1D-8 | 2.356377 | 2.83024 |
| IGKV2-29 | 2.833118 | 2.196068 |
| IGKV2D-30 | 3.823649 | 3.226753 |
| IGKV2D-40 | 3.189176 | 3.264525 |
| IGKV3D-11 | 3.003446 | 3.356605 |
| IGLC2 | 3.500068 | 2.197444 |
| IGLC3 | 3.552097 | 2.145453 |
| IGLC7 | 2.06125 | 2.989453 |
| IGLV1-36 | 2.914544 | 2.496547 |
| IGLV1-47 | 3.554114 | 2.053655 |
| IGLV2-28 | 2.231324 | 3.883846 |
| IGLV3-25 | 4.40631 | 2.714419 |
| IGLV3-27 | 4.625536 | 2.713079 |
| IGLV4-69 | 3.584065 | 2.636072 |
| IGLV7-43 | 2.275896 | 2.483539 |
| IGLV7-46 | 3.745818 | 2.71555 |
| IGLV9-49 | 4.235036 | 3.622747 |

| | | |
|---|---|---|
| ITGA2B | 3.041642 | 2.261747 |
| ITGB3 | 2.47009 | 2.555719 |
| KCNT2 | 2.911728 | 4.869364 |
| LINC00958 | 2.17638 | -4.88715 |
| LINC01565 | 2.619874 | -2.6372 |
| LOC101928932 | 4.065671 | 2.805649 |
| LOC105370027 | 2.035779 | 3.509397 |
| LPAR4 | 2.474683 | 2.008761 |
| MAGI2-AS3 | 2.468627 | 2.392191 |
| MMP1 | 2.934984 | 2.353589 |
| MMRN1 | 2.196398 | 2.154733 |
| MYBL2 | -3.35656 | -2.07901 |
| MYL4 | 3.101892 | 2.502675 |
| MZB1 | 3.581419 | 2.183014 |
| PBK | -3.99206 | -2.31159 |
| PF4 | 2.02631 | 2.240103 |
| PF4V1 | 2.893831 | 3.648445 |
| PPBP | 2.221363 | 3.340847 |
| PROS1 | 2.179366 | 2.09888 |
| PTPRN | 2.040823 | 3.150159 |
| PYCR1 | 3.102169 | 2.508068 |

| | | |
|---|---|---|
| RNU4-38P | 2.753866 | -2.48801 |
| SDC1 | 5.054987 | 3.499158 |
| SMIM5 | 2.164395 | 2.398299 |
| TEX15 | 3.180105 | 4.852666 |
| TK1 | 3.130801 | 2.184802 |
| TNFRSF17 | 3.310378 | 2.175281 |
| TNNT1 | 2.989855 | 2.708745 |
| TNNT2 | 2.05359 | -4.6063 |
| TREML1 | 2.884952 | 2.318918 |
| TRPM6 | 2.529528 | -2.40477 |
| TTK | 2.439725 | 2.944328 |
| VEGFC | 2.019395 | 2.274076 |
| ZFPM2 | 2.219203 | 3.246874 |

14
